# Describing the Ecology of Parenting Based on Preschool Mothers’ Social Relationships in Korea: An Ecological Theory Approach

**DOI:** 10.3390/ijerph192315864

**Published:** 2022-11-29

**Authors:** Kaka Shim, Hyunsook Shin

**Affiliations:** 1 Department of Nursing, Sangmyung University, Cheonan-si 31066, Republic of Korea; 2 College of Nursing Science, Kyung Hee University, Seoul 02447, Republic of Korea

**Keywords:** social network, ecology theory, parenting of preschooler

## Abstract

Mothers’ social networks are important to their children’s health but still remains poorly understood in Korea. The purpose of the study was to explore the elements of social relationships in Korean preschool mothers to describe their parenting ecology. Data were collected from interviews with 32 mothers according to the Social Network Interview questionnaire and from their seven-day diaries describing their daily social relationships. Eleven ecological elements were identified. The social relationships of these mothers involved seven types of people: relatives, mothers of their children’s friends, kindergarten and learning center teachers, domestic workers providing parenting support, husbands, friends, and children’s friends. The mothers’ social relationships were also associated with four location types: kindergartens, private learning centers, health services centers, and private entertainment centers. The strongest element of the ecology of parenting in terms of frequency was relatives, followed by the mothers of their children’s friends and kindergarten teachers; for the location, the strongest was kindergartens, followed by private learning centers and health services centers. In conclusion, the strongest elements in parenting ecology should be evaluated during the assessment of children’s growth and development, and incorporated in the assessment tools used.

## 1. Introduction

With various definitions in play, social relationships and their associated measurement methods are often in conflict [1,2]. In general, however, a social relationship involves a network of people who are connected to and interact with an individual in some way. Scientifically, social relationships influence human health through different mechanisms [3,4]; therefore, properly intervening in social relationships might help improve an individual’s health. Regarding the health of children, the influence of social relationships begins in the preschool period and includes the relationships of the mother. In fact, children’s growth and development are directly related to the mother’s social relationships, thereby affecting the child’s overall health.

Spontaneous social interactions involving the communication of shared experiences are helpful for mothers to build social relationships [5]. These relationships can provide both direct and indirect support for parenting. Previous research has shown that the greater the number of social relationships of mothers and the greater their social support, the more positive their interactions with their children, the less parenting burden they feel, and the greater the personal happiness they experience. Hence, social relationships not only connect mothers to a wider society, but also help them adapt to their maternal responsibilities. By establishing a social network, mothers can readily access useful information and share knowledge of mothering with other mothers who have similar experiences [6]. Though children are clearly influenced by their own social interactions with other children and certain adults, the social relationships of the mothers including their interactions with spouses, relatives, friends, and neighbors can have indirect but critical effects on the health of children. Thus, relationships having a direct impact on a mother’s role as a primary caregiver require consideration.

Nowadays, most Korean families are nuclear. As a result, the family support system is reduced, leading to difficulties such as the lack of substitute parenting from grandparents and other relatives [7]. Considering the challenges of parenting with little or no family support, women may rather avoid marriage and childbirth so they will not need to stop pursuing their careers. For Koreans who choose to marry and have children, the preschool years—the period when children begin to exercise social independence and personal autonomy—are a particularly challenging time because of the diminished family support [8]. When children attend preschool or kindergarten, the social relationships of Korean mothers tend to expand greatly as their children’s own social relationships rapidly develop. The mothers’ diverse social relationships such as with teachers or with other children’s mothers influence their care for their preschool children and subsequently the children’s social development and health [8]. The mothers’ social relationships come to constitute one of their parenting functions and carry parenting meanings that affect the children’s life circumstances, needs, expectations, and resources. Earlier studies emphasized the effects of social context on parenting, mostly by investigating and analyzing the characteristics of the mothers’ relationships with the surrounding society [9,10,11].

Bronfenbrenner’s [12] ecological model can explain the social networks of the mothers of preschool children. Bronfenbrenner asserted that health-related human behavior is influenced by the entire environment, especially through the individual’s social interaction [13]. In light of the assertion of ecological system theory that everyday experience is the greatest determining factor in the development and health of children, and given that mothers are generally the primary caregivers, the mothers’ social relationships and associated experiences should be carefully examined to identify potential means of supporting the health development of children [11].

The relationship of children’s individual characteristics to their mothers’ social networks has already been studied globally. The mother is affected by her own characteristics such as satisfactory feelings or stressful experiences related to parenting, but also by the nearest family environment around her (the microsystem level) including her partner and significant others [14]. For example, Heath et al. [15] reported that the maternal network during the preschool years strongly affected the mothers’ thinking and behavior and had an indirect but powerful impact on their children’s health and development [16]. The social relationships of mothers of preschool children differ greatly from those experienced during their children’s infancy, when the mother’s social network tends to be relatively small. During the preschool years, the child’s growing interaction with the social environment necessarily changes the nature of parenting [17]. Considering the transitional nature of the preschool years, comprehensively examining the mothers’ ecology of parenting during this period is crucial [18].

Although various phenomena related to parenting during the preschool period have already been identified, the ecology of parenting with respect to the mothers’ social relationships should also be defined using ecological theory as an analytical framework. Moreover, despite the evident importance of the mothers’ social relationships to child development and health [19], the relevance of the ecology of parenting for children’s health to the influence of the mothers’ social networks on their children’s health still remains poorly explored in Korea. Therefore, this study aimed to explore the social relationships as elements of ecological theory to describe the ecology of parenting for the mothers of preschool children in Korea.

## 2. Methods

### 2.1. Research Design

This study employed a qualitative content analysis design to identify ecological elements involved in the social networks of Korean mothers of preschool children. A qualitative method was chosen because it allowed the mothers to express their thoughts and feelings about the preschoolers’ ecological and environment through their social networks.

### 2.2. Sample and Setting

Participants with various backgrounds and from various regions of Korea were selected by purposeful sampling to achieve the richness and novelty of conceptualization. To recruit participants, a snowball sampling was employed in which one mother introduced the researcher to another mother, and so on. Participants with various backgrounds and from various regions of Korea were selected by purposeful sampling to achieve the richness and novelty of conceptualization [20]. Under the study inclusion criteria, participants were required to be mothers having at least one child aged four to seven years attending preschool and kindergarten institutions (with public and private) that reside in the center in Seoul, particularly the Kyung-gido city regions. After being informed about the purpose and procedures of the study, eligible mothers were invited to participate.

### 2.3. Data Collection

Participants were recruited after the study was approved by the institutional review board of a university in Korea. During the interview, participants learned the purpose and procedures of the study as well as their right to withdraw from the study at any time, before providing written informed consent. All participants were assured that their information would remain anonymous and would be permanently erased once the study was completed.

Study data were collected using semi-structured one-on-one interviews (Social Network Interviews [SNIs]) and parenting diaries from November 2015 to March 2016. The interviews were conducted using Cochran, Larner, Riley, Gunnarsson, and Henderson’s [21] questionnaire designed for SNIs. This instrument has been proven suitable for populations with diverse cultural and educational backgrounds, family structures, and social environments. It includes questions about (1) the names of members of the social network; (2) the characteristics of network members and their relationship to the respondent; (3) the exchange of social support with the members; and (4) the intensity of the relationships. The interviews were mostly conducted in the mothers’ homes, though some took place at their workplaces. The time needed to complete the interviews ranged from 60 to 80 min.

After the interviews, the participants were asked to complete online parenting diaries to further characterize their social relationships. Each mother kept a parenting diary of all contact (in person, by telephone, or by electronic messaging) with their social network members and also recorded interactions with other individuals, socioeconomic environments, and institutions during daily life for one week. Additionally, these mothers recorded all of the day’s interactions with network members every night before going to bed.

An online system was used to facilitate the completion of parenting diaries and associated communication between the participants and the researcher. The parenting diaries were written based on the Social Network Interviews questionnaire. Each evening for 7 days, the participants accessed their individual parenting diaries and completed them online for recording convenience. It included diary contents including: (1) Write down the person you met or called with the person related to your child (e.g., meeting with kindergarten teacher at school, phone with academy teacher, etc.); (2) Write down visiting a place with children, a place related to children, or a place related to a child; (3) Write down the activities with your child of the day; (4) Write down the method communicated with your children or with someone related to your children; (5) Other (freely write content related to children or feelings of parenting children). The following morning, the researcher reviewed each diary entry for clarity and separately recorded the entry’s parenting satisfaction score. The researcher telephoned the participant to clarify any ambiguous or incomplete contents and then recorded this telephone interaction with the participant in the online diary system. The participants could also use the online system to telephone or text-message the researcher when they had questions or needed guidance in completing their diary entries. All 32 mothers completed the 7-day diaries.

### 2.4. Data Analysis

The interview and diary data were examined by content analysis to identify ecological elements and their frequencies. Content analysis is widely used because it can achieve both understanding and knowledge of the study participants by quantifying the information they express orally or in writing [22]. In this study, the content analysis included active reading, verification, correction, modification, and the organization of data. After transcribing the audio-recorded interviews and downloading the diary contents, we read the transcripts and diary contents several times to fully understand all of the study data. We used words as the unit of analysis and categorized meaningful words and phrases from the interviews and diaries [23]. To ensure that no meanings were lost or ignored, we organized and coded the data during several interpretative steps [24]. The data were analyzed manually (without the use of computer). Based on the ecological theory, frequent microsystem elements were identified in the data.

We are familiar with the concepts of ecological theory and have substantial experience in content analysis based on coding standards. During coding, researchers have independently reviewed the interview transcripts and diary entries. After repeatedly reading the interview and diary contents, researchers collected similar meaningful words from sentences and coded them as one representative word. In the content analysis, they discussed any ambiguities in the coding process and problems with word interpretation. The coding discussion continued until the inter-rater reliability rate was above 90%. As the content analysis proceeded, the collected data were categorized according to the microsystem concepts of the ecological theory. The categories were repeatedly reviewed, discussed, and agreed upon by the researchers. Considering that participants used widely varied words and expressions in the interviews and diaries, words and phrases expressing a similar concept fell into the same category.

### 2.5. Methodological Rigor

In applying ecological theory, the meaningful parenting and cultural information have to be extracted from the interviews and diaries of Korean mothers. To achieve methodological rigor with respect to the qualitative data and minimize excessive translation, we only translated the ecological elements, meaningful units, and selected quotations for the results section. A bilingual English and Korean speaker with a master’s degree education translated the elements, units, and quotations. Another research team member who is a monolingual English speaker reviewed all of the translated data. In addition, two nursing faculty members who are bilingual and can fully understand Korean culture verified the adequacy of the data translation by comparing the themes, subthemes, and selected quotations written in Korean and English.

Furthermore, the study team’s research experience with the Korean population enhanced the study’s credibility. Regarding transferability, the study provided adequate descriptions of the research context, participant selection, data collection, and data analysis. Finally, confirmability was achieved through the repeated review of the qualitative data by two coders to verify the coding as well as the review of direct quotations from the transcribed text by two nursing faculty members to ensure the coding’s accurate representation of the participants’ information.

## 3. Results

### 3.1. Participant Characteristics

The mean age of the participants was 37.1 years, and that of their preschool children was 6 years (range: 5–7 years). Most families had one to three children (mean: 2.0). Of the 32 participants, 18 (56.2%) worked outside the home, and 14 did not have a job. Table 1 summarizes the characteristics of the study participants and their preschool children.

### 3.2. Ecological Elements

The content analysis of the interviews and diaries revealed 11 major categories representing the elements of the ecological theory in the social relationships of the Korean mothers of preschool children. The ecological elements were mainly divided into people and places. The people involved in the mothers’ social relationships were (1) relatives, (2) children’s friends’ mothers, (3) kindergarten and private teachers, (4) friends, (5) domestic workers providing parenting support, (6) husbands, and (7) children’s friends. The other ecological elements, representing the places where social relationships developed, were (1) kindergartens, (2) private learning centers, (3) health services centers, and (4) private entertainment centers. Table 2 lists the ecological elements along with the meaningful unit to support each element, and their frequencies of occurrence in the overall qualitative data.

### 3.3. People Involved in Mothers’ Social Relationships

*Relatives.* Relatives are defined as being linked by blood or marriage. In Korea, the children’s grandparents are important caregivers and advisors in the ecology of parenting. Although the data included many references to the mothers’ parents, the category of relatives also included the mothers’ siblings, parents-in-law, aunts, and cousins. Mothers reporting relatives as a microsystem narrated some pressures from relatives including mother in-laws and their families of origin.

*Children’s friends’ mothers.* The mothers’ social relationships with the mothers of their children’s friends generally focused on their children’s activities.

*Kindergarten and private teachers*. In addition, Korean mothers frequently connected with kindergarten teachers in both public and private facilities and with private teachers in learning centers and home-schooling situations. Considering their children’s many activities and private tutoring, the participants connected with many types of private teachers. The researchers classified all the teachers mentioned in the interviews and diaries as either kindergarten teachers or private teachers—mothers called them all “teachers”.

*Friends*. As mentioned, most mothers had formed friendships with many of the mothers of their children’s friends through their children. However, in this study, friends were defined as the mothers’ personal acquaintances, peers, and work colleagues.

*Domestic workers providing parenting support.* Furthermore, most mothers employed domestic workers providing parenting support including nannies to look after the children and housekeepers to do the cleaning and laundry. Of note, Korean mothers used the word “aunt” to refer to both female relatives and paid caregivers. Thus, the researchers carefully examined the meanings of “aunt” in various specific contexts and classified aunts as either “relatives” or “domestic workers”.

*Husbands.* The husband element clearly referred to the participants’ spouses; the participants used many different expressions to refer to their spouses including their names, nicknames, and terms of endearment.

*Children’s friends.* The last element in the people category was children’s friends. For example, some mothers said that their children’s friends had visited their homes for playdates or sleepovers; during these activities, the mothers would spend several hours looking after and interacting with these children, thereby forming relationships with them.

### 3.4. Places Social Relationships Occurred

*Kindergartens and private learning centers.* As to the places constituting ecological elements, kindergartens and private learning centers were the most frequently mentioned locations. Regardless of whether the children attended public or private facilities, the term “kindergarten” referred to locations that the participants’ children visited daily. All other learning, study, and developmental facilities were classified as private learning centers. Many mothers took their Korean preschool children to private learning centers, and some of them said that they connected with other mothers to exchange parenting information.

*Korean health services centers*. Korean health service centers offer easy access to primary care, and the participants took their children to these facilities for minor medical and dental treatment or for growth and development screening.

*Private entertainment centers.* As the final ecological element, private entertainment centers were regarded by some mothers as indoor play facilities where they took their children and interacted with other mothers.

### 3.5. Most Frequent and Dependable People and Places in Social Relationships

The most frequently presented ecological element was relatives, followed by the mothers of their children’s friends, kindergarten teachers, and private teachers. In terms of place, kindergarten was the most frequent, followed by private learning centers and then health service centers. Table 3 shows the strength of the ecological elements retrieved from the interviews of the mothers. Among the ecological elements, the mother’s own mother, husband, relatives, and the mothers of their children’s friends were the main social relationships within the networks that they built and used to care for their preschool children.

## 4. Discussion

Based on Bronfenbrenner’s ecological theory, this study described the ecological elements of parenting revealed by the social relationships of Korean mothers of preschool children. Bronfenbrenner defined an “exosystem” as “one or more settings that do not involve the developing person as an active participant, but in which events occur that affect, or are affected by, what happens in the setting containing the developing person” [13]. The “microsystem” was also defined as the groups and institutions that most immediately and directly impacted child development including the family, school or daycare, peer group, and community environment [12]. The present study identified 11 elements of the microsystem and exosystem of the ecology of parenting.

Relatives constituted the strongest ecological element, consistent with previous Korean research results. For example, in an ecological study of parenting stress among Korean working mothers, blood relatives were the strongest ecological element [8]. The present study specified that among the blood relatives, grandmothers (77%) had the strongest relationship to parenting. Thus, relationships with blood relations are an important social relationship element in the mothers’ parenting ecology [25]. As in our study, blood relatives have traditionally been viewed as an important factor in parenting ecology in Korea; if they are absent and no one in the social system is available to replace them, an additional parenting burden occurs.

For nonrelatives, the mothers of their children’s friends exerted the greatest impact on parenting ecology. Mothers tend to establish close relationships with the mothers of their children’s friends so that they can share information related to the children and take care of each other’s children [26]. This phenomenon is especially prevalent among preschool mothers. As their children begin a new and diverse social life with the development of social relationships, mothers also acquire a new social network [27]. In the past, such social relationships naturally began and grew in public play-spaces such as small neighborhood streets, community parks, and school playgrounds. However, mothers nowadays feel compelled to invite their children’s friends into their homes or take them along to entertainment centers or museums for their children’s social development. Through these activities, new interactions and relationships with other mothers are formed. Consequently, parenting experience and advice obtained from the children’s grandparents in the past can now be acquired through relationships with the mothers of their children’s friends [28].

Children’s social relationships begin to expand when they start preschool or kindergarten, at 4 to 7 years of age [17]. Our study found that the mothers’ relationships with kindergarten teachers formed one of the strongest ecological elements. The mother monitored and confirmed the child’s overall development through her relationship with the teacher, indicating that the interactions between the kindergarten teacher, mother, and child are important to child-health development. Hence, the mother’s new relationship with the teacher becomes an important part of parenting ecology [7] and functions like a mesosystem in ecological theory. Mothers connect with teachers not only in person but also through telephone use and online applications for school announcements, text messages, and the like. Thus, in contemporary society, technological means can help maintain and strengthen the mothers’ relationships with kindergarten teachers as well as other mesosystem elements.

Outside the kindergarten context, preschool children in Korea commonly undergo private tutoring and meet with various private teachers both in private learning centers and at home. According to the Korea Statistical Office [29], 60% to 70% of preschool children receive private tutoring in the arts, music, and physical education. Moreover, private teachers provide more than half of Korean language, mathematics, and English instruction. In Korea, it is a social phenomenon because the parents’ income, time, and energy are allocated to achieve a high educational achievement of children, and it is considered as one of the most important roles of mothers to ensure their children’s educational achievement [30]. Consequently, mothers are increasingly connected with such educators and inevitably form close social relationships with them, influencing their parenting ecology.

In addition, the mothers’ social relationships included several friends with whom they shared their thoughts regarding parenting, consistent with a previous study [31]. Over time, the mothers maintained a continuous and intimate relationship with these friends through telephone calls and text messages. The more mothers interact with friends, the greater the socialization of their children and the more friends they associate with [32]. Brown and Harris [33] also found that mothers with broader social relationships had warmer and less intrusive interactions with their children. In our study, mothers who maintained good social relationships with friends perceived that their children showed an improved social competency. Therefore, structured community support for mothers to establish a healthy network of friends would benefit not only the mothers, but also their children.

A recent Korean survey on parenting revealed that parenting support received from nonrelatives is increasing [29], partially because of the lack of relatives living nearby. In our study, domestic workers provided substantial parenting support by serving as babysitters and housekeepers, either part-time or full-time. The need for domestic workers to provide parenting support grows as the number of working women increases; thus, domestic workers become involved in the parenting ecology. Our participants maintained close contact with their domestic workers via electronic devices and in person because they became quite dependent on these helpers to contend with the challenges of parenting.

Moreover, the level of interaction between mothers and their husbands was relatively low. Gross, Fogg, and Tucker [34] reported that despite increased societal interest in the parenting by fathers, fathers were less likely to participate in the study intervention. In Korea, family dialog tends to be minimal; thus, as expected, the mothers’ parenting-related interaction with their husbands was less frequent in our study. The father’s involvement in childcare may buffer the effect of maternal psychological distress, or it could decrease a mother’s perception of negative family functioning [35]. Hence, Korean families need systematic policy support and programs that promote social interaction between spouses and within families. To increase their participation in the Korean ecology of parenting, fathers need to assume a greater parenting role within their families and to engage in increased social interaction with their wives [36].

Mothers also served as moderators between their children and their children’s friends. For example, they helped their children make friends, arranged playdates when their children could engage with peers, and provided transportation to meetings outside the kindergarten environment. They also influenced interactions during playdates by teaching their children and children’s friends how to initiate peer activities, structuring the play setting, and regulating the frequency of their playdates [18]. In doing so, the mothers themselves formed social relationships with their children’s friends. Along with providing their children with opportunities to expand their own social networks, the mothers’ connections with their children’s friends may foster a greater commitment or link to conventional social involvement.

Regarding the locations of the mothers’ social relationships, kindergarten was the most frequently mentioned, followed by private learning centers, health services centers, and private entertainment centers. The latter three locations particularly reflect the Korean ecology of parenting, given the country’s emphasis on children’s education and health, and its shortage of public playgrounds. Many Korean parents believe that supplementing their children’s school experience with additional tutoring at private learning centers is necessary [37]. Moreover, Korean mothers have easy access to hospitals and clinics and frequently take their children to health care providers for health checkups, health information, and treatment for minor illnesses. The lack of public play facilities also leads Korean mothers to visit private entertainment centers to encourage their children to interact and play with others.

In summary, Korean mothers have multiple social connections with people and places that affect their parenting. We characterized 11 categories of relationships as ecological elements of the Korean ecology of parenting. Our findings highlight the changes in parenting ecology resulting from Korea’s evolving economy, culture, and social environment. Correspondingly, Korean mothers perceive the need for an expanded parenting support system consisting of modern parenting guidelines, information on community resources for families, and, for working mothers, greater access to affordable daycare facilities.

## 5. Limitations

As a future study on children’s health, we need to analyze not only the microsystem, but also the more extensive systems in Korea’s complex parenting system. Based on this results, it is necessary to examine the relationship between the parenting ecological factors and demographic characteristics, social relationships, and the environment in detail, and it is necessary to conduct in-depth investigation into the participation and support of family members, except for the mother. In addition, this study was limited to representing the national profile of participants that is the small, non-representative sample size of participants. Considering its qualitative approach, future studies have to need a sample size based on the sociodemographic profile of participants relative to the national profile. However, the most powerful parenting ecological factors found in this study should also be considered in tools used to assess the growth and development closely related to children’s health.

## 6. Conclusions

This study is the first to apply ecological theory to the Korean ecology of parenting in the mothers of preschool children. Our findings revealed 11 distinct elements of parenting ecology. Most of the mothers’ social relationships focused on interactions with people and places that they regarded as important to their parenting. Given that the mothers’ social relationships generally influence their children’s health, growth, and development, our study confirmed the significance of Korean mothers’ parenting ecology. To support future application of the relevance of the ecology of parenting for children’s health in Korea, modifications should be made based on the realities of Korean parenting ecology. Our findings will lead to research on intervention strategies that can effectively educate Korean parents about the parenting supports and resources available to Korean parents including medical, educational, and nursing services.

## Figures and Tables

**Table 1:** Characteristics of study participants and their preschool children (*n* = 32).

Characteristic	Classification	*n* (%)
Childbirth order ^a^	First child in preschool	20 (62.5)
	Second child in preschool	11 (34.3)
	Third child in preschool	1 (3.1)
Child’s preschool	National public kindergarten	1 (3.1)
	Private kindergarten	5 (15.6)
	National public daycare center	1 (3.1)
	Private daycare center	5 (15.6)
	Private academic center	20 (62.5)
Mother’s highest education level	High school	1 (3.1)
	University or college	18 (52.2)
	Master’s or doctoral degree	13 (40.6)
Mother’s employment status	Yes	18 (56.2)
	No	14 (43.7)
Area of residence	Seoul	15 (46.8)
	Gyeonggi-do	9 (28.1)
	Other regions	8 (25.0)

Note. ^a^ Five participants each had two children attending preschool.

**Table 2:** Meaningful units, frequencies of occurrence, and ecological elements *n* = 1718 (%).

Category	Meaningful Units	Microsystem Element	Number of Observations (Frequency)
Relationships with People	My mother, mom, our mother, children’s grandmother, younger sister, my brother, older sister, older brother, mother-in-law, grandmother, my aunt, female cousins, father-in-law, brother-in-law	Relatives	310 (18.0)
	Kindergarten friend’s mom, daycare center friend’s mom, mother of friend from previous daycare center, children’s playschool friend’s mother, children’s friends in postpartum care center, children’s friends in learning center, children’s friend’s mom in church	Children’s friends’ mothers	293 (17.1)
	Our teacher, daycare center teacher, children’s teacher, class teacher, our associate teacher	Kindergarten teacher	270 (15.7)
	Home-school teacher, assistant teacher, foreign teacher, shuttle teacher, safety teacher, consultant teacher, art teacher, piano teacher, lecture teacher, reading/thinking teacher, cultural center teacher, swimming teacher, soccer teacher, ballet teacher, mathematics teacher, Kumon teacher, Montessori teacher, pottery teacher, Baduk teacher, violin teacher, tutor teacher	Private teacher	190 (11.1)
	My friends, church friends, friends in the neighborhood, school alumni, college friend, my brother whom I know, Facebook friend, yoga center friend, fitness center friend, friend I met in dance class, my boss, my colleague, working sister, work comrade, partner, work buddy, chief, team leader, senior comrade, friend whom I can tell secrets, friend who calls every day, friend I am more comfortable with than my husband, best friend	Friends	80 (4.7)
	Babysitter, housekeeper, homemaker, aunt, cleaning assistant, working grandmother, housekeeping assistant, aunt who comes once a week, a part-time aunt, a nanny, Filipino, immigrant nanny, caregiver	Domestic workers providing parenting support	79 (4.6)
	Husband, groom, baby’s dad, my groom, spouse, brother, my house uncle, honey, piggy, brother, sister, baby, youngest, older brother, my baby, my puppy	Husband	62 (3.6)
	A classroom friend like in kindergarten, a child’s friend from a former daycare center, a friend in playschool, a friend in a culture center, a friend in a neighbor’s apartment, a daughter of my friend	Children’s friends	49 (2.9)
Locations of Relationships	Kindergarten, A *, B *, public daycare center, C *, Yujeong Kindergarten, English Kindergarten, private daycare center, playschool, daycare center, private kindergarten	Kindergarten	189 (11.0)
	YMCA, Taekwondo, science center, Baduk academy, board-game school, mathematics institution, welfare center, swimming center, gym center, department-store center, childcare information center, town hall library, children’s libraries, local children’s library	Private learning centers	117 (6.8)
	Neighborhood pediatrics clinic, university hospital, pediatrics, dentistry, otolaryngology, dermatology, emergency room, ophthalmology, nearby clinic, children’s dentistry clinic, internal medicine clinic	Health services centers	40 (2.3)
	Kids’ cafe, indoor playground, giraffe indoor play place, imaginary country, outdoor/indoor playground, apartment complex playground park, indoor experience play place	Private entertainment centers	39 (2.3)

*: Name of a kind of private kindergarten.

**Table 3:** Strength of the ecological elements.

Strength	Ecological Elements					
1st element *n* = 32 (%)	Mom’s mother	Husband	Relatives	Kindergarten teacher	Children’s friends’ mothers	Domestic workers providing parenting support
	9 (28.1)	7 (21.3)	6 (18.7)	5 (15.6)	3 (9.3)	2 (6.2)
2nd element *n* = 31 (%)	Mom’s mother	Husband	Relatives	Kindergarten teacher	Friends	Children’s friends’ mothers
	10 (35.4)	6 (19.3)	4 (12.9)	3 (9.6)	2 (6.4)	2 (6.4)
3rd element *n* = 30 (%)	Relatives	Children’s friends’ mothers	Domestic workers providing parenting support	Friends	Mom’s mother	Private teacher
	9 (26.6)	8 (26.6)	5 (16.6)	3 (10.0)	2 (6.6)	2 (6.6)
4th element *n* = 20 (%)	Children’s friends’ mothers	Husband	Relatives	Kindergarten teacher	Mom’s mother	Private teacher
	6 (30.0)	4 (20.0)	3 (15.0)	3 (15.0)	1 (5.0)	1 (5.0)
5th element *n* = 18 (%)	Friends	Children’s friends’ mothers	Relatives	Husband	Friends	Kindergarten teacher
	5 (27.7)	4 (20.0)	3 (22.2)	2 (11.1)	1 (5.5)	1 (5.5)

## Data Availability

The data are available upon reasonable request from the corresponding author.
